# Experiences and Perspectives of Families of Psychiatric Hospitalisation of Their Adult Family Member: A Qualitative Systematic Review

**DOI:** 10.1111/inm.70042

**Published:** 2025-07-09

**Authors:** Jane Karpa, Amanda Kenny, Andrea Thomson, Marian Ramage, Monica Ginn Forsyth, Kendra Rieger

**Affiliations:** ^1^ Faculty of Health Studies Brandon University Winnipeg Manitoba Canada; ^2^ Lincoln International Institute for Rural Health, University of Lincoln Lincoln UK; ^3^ Violet Vines Marshman Centre for Rural Health Research, La Trobe Rural Health School La Trobe University Melbourne Victoria Australia; ^4^ Professor Emerita, College of Science Health and Engineering La Trobe University Australia Australia; ^5^ Faculty of Health Studies Brandon University Brandon Manitoba Canada; ^6^ John E. Robbins Library Brandon University Brandon Manitoba Canada; ^7^ Brandon University Brandon Manitoba Canada; ^8^ School of Nursing Trinity Western University Langley British Columbia Canada

**Keywords:** carers, family, family‐centred, hospitalisation, mental health, psychiatry

## Abstract

Families who encounter the hospitalisation of an adult family member for a serious mental illness are frequently excluded from treatment and care planning. It has been established that family involvement is beneficial and positively impacts health outcomes; however, health systems continue to constrain collaborative practices. The aim of this qualitative systematic review was to explore families' views of their experiences of psychiatric hospitalisation of their adult family member and the barriers and benefits for the uptake and delivery of collaborative relationships between mental health clinicians, families, family carers, and consumers. In September 2022, systematic searches were conducted for primary studies in: CINAHL, PsycInfo, PubMed, Web of Science, and dissertations in ProQuest Dissertation and Theses Global. The searches were updated February and September 2024. Retrieved articles were managed in Covidence and screened by three reviewers at the title and abstract and full text stages. Following screening, a total of 27 articles formed the review dataset. Study quality was assessed using the well validated Critical Appraisal Skills Programme checklist. Three themes emerged from the review: (1) Impenetrable System—‘*Expert‐itis’*; (2) Perpetuating Family Invisibility; and (3) Unlocking the Door. Evidence consistently described participants' experiences of inhumane professional attitudes and practices in hospital mental health services. Further research on the types of interventions and communication strategies to promote family engagement and involvement in mental health service design and delivery is needed.

## 1 Introduction

Family involvement in facilitating the mental health recovery of a family member is important (Clarke and Winsor 2010). Shared decision‐making with families has been directly linked with positive health outcomes (Huang et al. 2019). However, families are frequently excluded from treatment and care planning. Reasons for family exclusion include concerns of confidentiality among mental health professionals, lack of education or skill in working with families (Goodwin and Happell 2006), or hesitancy when family members are viewed as barriers to the consumer‐health clinician relationship (Creasy et al. 2015).

Further barriers to family inclusion have also been recorded. In a study exploring the experiences of family carers of consumers in an acute psychiatric or chronic psychiatric rehabilitation ward, Hsiao et al. (2019) found that the provision of general and specific information was inadequate compared to a coordinated partnership that provided respectful and comprehensive care. Health professionals have stated that a lack of collaborative relationships is because families do not always know what they should ask for or expect (Denham et al. 2016). Families of people with mental illness want to be involved in all aspects of care; however, the health system continues to restrict their involvement in the planning, delivery, and evaluation of care (Fleming et al. 2012; Levack et al. 2009; Miller 2012; Stejskal 2012).

In 2017, Doody et al. stated their integrative review was the first to explore family experiences of care planning in adult mental health services. They included 15 articles in their review (seven qualitative, seven quantitative, and one mixed method) from different countries (United Kingdom, Sweden, Ireland, the United States of America, Norway, Italy, and Israel). Their focus was on any adult mental health services and was limited to family involvement in care planning. The findings indicate a wide divide between family members and service providers, with collaborative decision‐making thwarted by a lack of communication, uncoordinated care planning, constraints blamed on confidentiality, and claims of insider knowledge (Doody et al. 2017). Evidence from more current reviews reports caregivers of mental health inpatients are consistently experiencing negative experiences with inpatient services (voluntary, involuntary, forensic) and emergency services (Lavoie 2018; Abou Seif et al. 2022; Stuart et al. 2020; Sugiura et al. 2020; Vestphal et al. 2023).

While previous reviews have focused on separating carer experiences between voluntary and involuntary inpatient services, this systematic review directly addresses this gap by focusing on qualitative research for an in‐depth exploration of families' experiences of voluntary or involuntary acute care hospital services including forensics. Seers (2015) stresses the importance of qualitative systematic reviews in understanding experience and uncovering new understandings that are directly applicable to practice. The aim of this review was to explore families' views of their experiences of psychiatric hospitalisation of their adult family member and the barriers and benefits for the uptake and delivery of collaborative relationships between mental health clinicians, families, family carers, and consumers. Synthesising the qualitative evidence of family members and family carers' experiences and perspectives on psychiatric hospitalisation of their adult family member will continue to inform international clinical practices, policy decision‐making, and future research.

### 1.1 Operationalising Terms

In this review, families were defined as those who they believe themselves to be: “a self‐defined group of individuals” (Eggenberger and Nelms 2007, 284). This could include parents, children, siblings, aunts, uncles, step‐members, and close family friends. The family carer was defined as the member most likely to assume these caregiver responsibilities and influence family functioning (Gan et al. 2006; Livingston et al. 2010). Hospitalizations were defined as shorter‐term acute psychiatric admissions or chronic psychiatric rehabilitation (denoted by longer stays in hospital). Families' experiences and perceptions related to any involvement with the hospital staff during the entire time of hospitalisation, encompassing discharges and transitions back into the person's home community. Mental illnesses were operationally defined as having a long‐term course and leading to significant social and occupational dysfunction including schizophrenia, schizophrenia spectrum and other psychotic disorders, suicidal behaviours, schizoaffective disorder, and bipolar disorder (Yin et al. 2020).

## 2 Method

General principles for conducting systematic reviews (Khan et al. 2003) and specific guides for conducting/reporting qualitative systematic reviews (Tong et al. 2012; Seers 2015; Butler et al. 2016) were followed to ensure rigour and replicability in the approach. Reporting was guided by the PRISMA 2020 statement, an updated guideline for reporting systematic reviews (Page et al. 2021), and the guidelines for reporting qualitative synthesis (Tong et al. 2012). The protocol is registered with PROSPERO (# CRD42022311568).

The six‐member research team brought rich and diverse perspectives to this review. The team included a university librarian, a master's graduate research assistant, and four experienced researchers from across Western Canada and the United Kingdom/Australia in the discipline of nursing/psychiatric nursing and the social sciences.

### 2.1 Literature Search

The search strategy was developed by the principal investigator (initials to be added following review) and the academic librarian team member (initials to be added following review). The intent of the strategy was to cast a broad net, with as much precision as possible, to ensure the capture of all relevant articles. Four concepts were established: person with an acute mental disorder, acute healthcare setting, family members of the person with the mental disorder, and experiences of the person's family members in the acute healthcare setting.

Key terms and phrases were identified by searching a core sample of relevant articles that had been identified in preliminary planning for this review. These terms were used to identify thesaurus terms, subject headings, and MeSH terms in the five databases searched: CINAHL, PsycInfo, PubMed, Web of Science, and ProQuest Dissertation and Thesis Global. In accordance with the recommendations of Lefebvre and Duffy (2021) for librarian or information specialist involvement in reviews, a Peer Review of the Electronic Search Strategy (PRESS) review was completed for both the CINAHL and PubMed database searches by two academic librarian colleagues before running the finalised searches in the remaining databases. (see Data S1 and S2) This allowed for input from two librarians not involved in the project to assess the search strategies and provide feedback to improve the completeness and comprehensiveness of the search strategies. In each database, the Boolean OR operator was used between terms within the concepts, and the concepts were combined using the Boolean AND operator. (see example in Table 1; complete search strategy details are contained in Data S3).

**TABLE 1:** PubMed search: Boolean operators OR and AND applied.

**Concept 1**: Person with an acute mental disorder
Mental illness OR mental disorder OR psychiatric disorder OR psychiatric illness OR mentally ill OR mental health condition OR mental health disorder OR mental health problem OR mental health issue OR psychiatrically ill OR Mental Disorders OR Mentally Ill Persons
AND
**Concept 2**: Acute healthcare setting
Emergency room OR emergency department OR emergency health service OR psychiatric hospital OR acute care OR acute inpatient OR emergency service, hospital OR Involuntary Commitment OR Commitment of Mentally Ill OR Emergency Medical Services OR Intensive Care Units OR hospitals, psychiatric OR Critical Care OR emergency services, psychiatric OR Mental Health Services OR Psychiatric Department, Hospital OR Involuntary Treatment, Psychiatric OR Psychiatric Nursing
AND
**Concept 3**: Family members of the person with the mental disorder
Families OR Family OR parent OR caregiver OR carer OR relative OR spouse OR adult child OR partner OR stakeholder OR mother OR sibling OR sister OR brother OR father OR wife OR wives OR husband OR daughter OR son OR sons OR grandparent OR grandmother OR grandfather OR Proxy OR proxies OR friend OR boyfriend OR significant other OR girlfriend OR elder OR loved one OR niece OR nephew OR cousin OR uncle OR aunt OR Friends OR Proxy OR Grandparents OR Siblings OR Spouses OR Family OR Parents OR Adult Children OR Caregivers OR Legal Guardians
AND
**Concept 4:** Experiences of the person's family members in the acute healthcare setting
Intervention OR attitude OR discharge plan OR shared decisionmaking OR family‐centred care OR care plan OR treatment OR need OR involvement OR collaboration OR perception OR perspective OR impression OR opinion OR experience OR health system response OR healthcare system response OR healthcare system response OR Psychosocial Intervention OR Attitude of Health Personnel OR Patient Discharge OR Decisionmaking, shared OR Family Nursing OR Patient Discharge Summaries

#### 2.1.1 Inclusion Criteria

Studies were included if they were primary qualitative studies describing experiences of family members or family carers of a family member who had been hospitalised with an acute mental disorder such as depression, schizophrenia, psychosis, and/or bipolar disorder. Only primary qualitative studies focused on adult psychiatric hospitalisation (identified as emergency, acute and or forensic hospital care setting) were included. The search was limited to studies published in English. A date limiter was excluded to collect all pertinent articles.

#### 2.1.2 Exclusion Criteria

Primary quantitative and/or mixed methods studies were excluded. Studies which examined family experiences with an adult family member hospitalised because of substance abuse, eating disorders, or dementia were also excluded as these health issues, even though they may affect mental health, are usually viewed as substantive and singular disorders on their own. As treatment collaboration between families and healthcare professionals is further advanced in paediatric clinical settings, studies in which a child was identified with a serious mental illness were excluded. Studies that were located within community healthcare settings were excluded as the focus of the review was on inpatient hospitalisations. Table 2 outlines the inclusion and exclusion criteria.

**TABLE 2:** Inclusion and exclusion criteria.

Criteria	Inclusion	Exclusion
Participants	Family members, family carers, or close others (considered to be part of a family unit) of an adult family member (over the age of 18) who was hospitalised with a mental disorder	All who didn't identify as family members Children as family members
Person hospitalised	Adults 18+ suffering with an acute mental health disorder (depression, psychosis, schizophrenia, bipolar)	Children under 18 Adults with issues of substance abuse, eating disorders, and dementia
Setting	Emergency or acute healthcare hospital setting including voluntary, involuntary and forensic services	Community settings and services
Study design	Primary, qualitative studies	Any other types of studies
Language	English	Non‐English
Date	All dates	None

### 2.2 Study Selection

The first round of database searches was conducted between June 6 and 8, 2022. An updated search was conducted February 24–26, 2024, to capture studies published after the first searches. Following the identification of included articles, the reference lists of these articles were perused for additional relevant studies. To ensure the comprehensiveness of this review, a revised CINAHL search was conducted September 2024. The CINAHL search strings were modified. First, the search string for Concept #4 (experiences) was replaced with a qualitative search filter. Secondly, CINAHL Subject Headings search strings for the remaining three concepts (person, setting, acute mental disorder) were changed—MM (major subject heading) to MH (subject heading), and the expander **(+)** was applied where it was available. This search resulted in the inclusion of one further article.

The research team used the Cochrane program, Covidence, to manage search results and screening. Titles and abstracts, and then full texts of potentially relevant articles, were independently screened by three reviewers (initials to be added following review). When discrepancies occurred, discussion occurred between the three team members until consensus was reached.

### 2.3 Data Extraction

The following data were extracted from the included studies: author(s), year, country, aim(s), sample population, theoretical framework, study method, major findings (family experiences and perspectives), and conclusion. This data was tabulated in excel (Table 3). Data extraction was undertaken by five members of the research team.

**TABLE 3:** Data extraction.

Authors (year), location	Aim	Sample	Theoretical framework	Study method	Family experiences and perspectives	Conclusions
Acres et al. (2022), Australia	To gain a greater understanding of carers (18+) perspectives on ED nursing practices when consumers with BPD are requiring crisis support	13 participants who identified as the primary carer for a person with BPD	Not identified	Qualitative descriptive design‐focus groups	Four main themes emerged: (1) challenges in accessing crisis support; (2) the need for communication; (3) stigmatising practices; (4) different levels of care	The tone of theses findings was mostly negative. While the ED was viewed as a frontline service which has the capacity to link with other speacilist mental health support, for this participation group these expectations were not met. There was a stark contrast between expectations and negative experiences. Participants often experienced poor communication, stigmatising repsonses, and barriers to involvement
Allan (2013), London, United Kingdom. Professional Doctorate thesis	To explore the lived experience of parents of male patients who have been detained under a hospital order with restrictions (section 37/41 Mental Health Act 1983), subsequently conditionally discharged into the community, and then recalled under the restriction order back to hospital	6 parents of 5 sons who had been conditionally discharged and subsequently recalled to hospital under the Mental Health Act	Interpretative phenomenology informed by critical realism. Interpretative phenomneological analysis based on symbolic interactionism	Semistructured interviews related to the admission, conditional discharge and recall phases. Interviews lasted 67–84 min	Four sub ordinate themes were developed: *Emotional impact* that included subthemes of hope and reappraisal, fear and loss and identify; *Responsibility* that included subthemes of parents assume responsibility, autonomy versus infantilisation. Progress towards independence, uncertainty as to who is responsible, and blaming and feeling blamed; *An impenetrable system* with subthemes shut door, engulfed and open door; and *The expert system* with subthemes professionals assumed expertise and not treated as equals. Parents reported a cyclic process of hope and disappointment and expressed feeling isolated and unsupported when trying to access help in an impentrable system. They felt marginalised	A major theme for parents was their feeling of exclusion from the clinical team. The study offers in‐depth and illuminating insight into the experience of parents in this situation. A number of recommendations have been suggested both in terms of direct work with parents, and also regarding how forensic services can collaborate with parents more empathetically and effectively
Askey et al. (2009), UK	Explore the views and experiences of carers, service users and professionals with regard to what carers of people with psychosis need from mental health services	Total of 56 participants—6 focus group discussions 3 for carers, 2 for professionals, 1 for servie users—supplemented by individual interviews with service users and carers–cares were self‐defined Total of 22 carers took part ages from 35 to 67	Non identified	Qualitative—focus groups and indiviudal interviews	Improving care for service users; improving carer involvement in care of their relative; emotional and practical support; to be treated with respect; information, education and training needs	Overarching finding—carers'needs are not being metservice users and carers needs are interconnected and should not be seen as separate
Buriola et al. (2021), São Paulo, Brazil	To identify the perceptions of family members about the care and organisation of a pyschiatric unit in a general hospital	13 family members. 11 female 2 male degree of kinship from parents to siblings and children	Descriptive and exploratory research. Analysis of data used Bardin's thematic content analysis methodological framework	Semi structured interviews focused on what it was like to have a family member in the unit, what was important when admitted, what could be improved on admission. Data collected on gender, age, profession, kinship, and time spent living with person with a mental disorder. Interviews lasted 20–40 min	Two major categories—(a) Relevance of environment and multidisciplinary team in the treatment of people with mental disorders—physical structure contributes to care process, importance of environment to overcoming fears and anxieties, interdisciplinary approach which encourages psychosocial inclusion and socio‐cultural reintegration, equality and equity in care provided. (b) Weaknesses in care dynamics as a support in interpersonal relationships—need for stronger focus on activity, need for careful bed management, continuity of care is impacted by constantly rotating staff. Relatives perceptions of service predominantly positive	It is concluded that the understanding of the family, as a co‐participant in the therapeutic process, can positively influence the strategies of care in the context of mental health and, consequently, provide equity, autonomy, right and exercise of citizenship to people with mental disorders
Clarke and Winsor (2010), Winnipeg, Canada	To examine the impact of a young person's hospitalisation on parents and to determine the parent perspectives on their emotional and practical support needs	10 parents (9 mothers, 1 father) of young people aged 18–25 recruited through local support group and snowball sampling. Half of the participants were aged 50–59 and half 40–49	Descriptive and exploratory study within a qualitative paradigm. Aguilera and Messick's (1986) crisis theory. Data analysis completed using Morse and Field's (1995) processes	Semistructured interviews focused on three areas: perception of hospitalisation, situational supports during hospitalisation, coping mechanisms during hospitalisation. Interviews lasted 1–2 h	Themes developed: *feeling relief about receiving a diagnosis, shock and disbelief associated with the diagnosis of a mental illness, isolation associated with the stigma of mental illness, feeling excluded during the discharge process, grieving for the loss associated with an altered future*. Participants support came from family, friends and support groups. Mental health providers were not perceived as helpful or supportive	To make the situation worse, emotional and educational support was often hard to come by. The stigma of mental illness made their dilemma a difficult one to discuss with friends and even with other family members. They further felt ignored by and invisible to the professionals caring for their children. Because parents need to be active members of the caregiving “team” as their child heads out on the road to recovery, early engagement between parents and mental health professionals is crucial. Furthermore, healthcare providers must recognise that “just another admission” for them is a profound life‐changing crisis for the patient and their family and, as such, represents the classic opportunity accorded to a crisis—the potential for disaster versus the potential for lifegiving growth
Crisanti (2000), Calgary, Canada	To describe mother's experiences with the involuntary hospitalisation of their adult child with schizophrenia	3 mothers of adult children with schizophrenia recruited through the Calgary Chapter of the Schizophrenia Society of Alberta. Time since child's diagnosis ranged from 12 to 20 years	Qualitative design using phenomenology. Data analysis followed guidelines of vanKaam (1969)	Interviews focused on four chronological events: prior to hospitalisation, admission process, hospital stay, leaving the hospital. Interviews lasted on average 2.5 h	Themes developed: *demeaning experience*—no‐one sought their opinions; *baffled*—perplexed with the commitment process; *victimised*—by the health system; anxious—what would happen to their child after parent died; *judged*—getting rid of the problem. Overall, mothers felt inadequately supported by the system with providers described as very negative	Overall experiences were negative. Mothers felt baffled, victimised, anxious, and judged
Finlay‐Carruthers et al. (2018), United Kingdom	To undertake a detailed examination of the experience of parents who have an adult child diagnosed with a mental illness receiving compulsory treatment and support in a forensic mental health unit	Six parents of adult children who were inpatients of a medium secure unit	Qualitative design using interpretive phenomenological analysis—Smith (1996) and Smith & Osborn (2003)	Individual interviews focused on making sense of the participants world	Themes developed: *Something's not right, the onset of mental distress*—terrified during repeated crisis and knowing something was seriously wrong; *It's a terrible battle, relating with professionals*—strained and invalidating relationship with health professionals; *A very sad fact of life, caring with no end in sight—*diagnosis bought grief and sorrow. The future is perceived as a mixture of hope, uncertainty and never ending with parents committed to doing whatever it takes to look after child. Parents describe onset of illness as overwhelming, frightening and confusing with experiences of violence. Interactions with services were described as a battle	Their lived experiences were characterised by strained relationships with mental health services. Caregivers felt that their needs and views were often ignored leaving them to care for their distressed and often behaviourally challenging loved one
Førde et al. (2016), Norway	“The project aims to explore ethical challenges related to coercion and involvement in care from the perspectives of staff, patients and next of kin” p. 2	36 next of kin to 33 ill family members p. 2 Father (1) Mother (13) Sister (2) Daughter (3) Female partner to male (1) age: 23–57	Not included	Focus groups—“study consists of three focus group interviews and one individual interview with next of kin to adults and three focus group interviews and one parent interview with parents to adolescents” p. 2	The study had four themes: (1) Life as next of kin: described informal caregiving responsibilites (before hospitalisation), a lack of social support, feelings of guilt, shame, and pressure (2) The importance of involved during serious mental illness and coercion: During hospitalisation, next of kin wanted the ability to share and recieve information with treatment team, the recongized the limits of confidentiality, but wanted education (rather than personal health information). They wanted increased communication. “Preserving or repairing family bonds, during and after aggravation of illness and hospitalisation, was one reason why NOK felt that their involvement was necessary” (p. 4) as family will provide support following hospitalisation. A lack of invovlement was described as offensive. (3) Experiences with inadequate involvement: The lack of involvement with healthcare staff was experienced as emotionally difficult and offensive, the system was described as “hostile and cold” (p. 4), This was especially difficult when their family member was admitted involuntarilly. (4) Descriptions about useful or good involvement: Described the importance of being seen as a human being. Open dialogue with healthcare team was found to be helpful. Found that there was a need for staff to taker responbility to communicating with family and involving family	Family involvement is still far from satisfactory. This fact is a threat to the health of the next of kin, to the patient and to an effective use of formal and informal healthcare. Our study shows that next of kin often are met in a way which adds to their anxiety, sorrow and guilt when their seriously ill family member is subjected to coercive measures
Giacco et al. (2017), United Kingdom	“aimed to assess the perspectives of all these three parties, i.e., patients, carers and mental health clinicians on how to improve carer involvement in inpatient settings.” p. 2	86 participants (31 patients, 22 carers and 33 clinicians) p. 3. Mean Age: Carers 51 (15.8) Mother 13 (59%) p. 4 Father 1 (5%) Wife 2 (9%) Partner 1 (5%) Sister 2 (9%) Daughter 2 (9%) Sister and daughter 1 (5%)	Not included	Focus groups‐separate focus groups were held with carers, patients and clinicians	Two themes were found with several subthemes. The authors noted congruence between patient, carer and clinician responses in themes. (1) “Carer involvement”—Carers should be involved as soon as possible into hospitalisation. Carers wanted to receive information, participate in decisions about care and discharge and receive emotional support by staff. When carers are involved, their personal knowledge of the patient's condition should be utilised. Carers reported that a lack of information was frustrating. They also wanted education on medication. (2) “Challenges to carer involvement” may included obtaining valid patient consent and there were barriers in sharing appropriate information. Noted an area of improvement would be on empathetic communication, even when patient does not consent to information sharing. (the second theme focused mainly on clinician responses, the information included was based on carer reports)	A framework for carer involvement in inpatient psychiatric care, resulting from our findings is reported
Hickman et al. (2016)	To better inform our understanding of the experiential impact of hospitalisation on the parents of young people with early psychosis, leading to recommendations that may improve the experience of hospitalisation for relatives and service users	Six parents of service users who had been hospitalised with early psychosis	Not included‐Interpretative Phenomenological Analysis used	Semistructured interviews focused on collecting participants' narratives of their experiences of their son or daughter's hospitalisation. Questions were open‐ended and enquired about the participants' experiences and sense‐making, and were specifically situated within the context of their experience	Five themes emerged from the data. Accepting and blaming, Feeling out of control, Hospitalisation as temporary containment, Feeling let down by services and Stigma. For theme 1: Participants attempted to make sense of the psychosis by taking a polarised position of either ‘acceptance’ or ‘blame’. For theme 2: Participants indicated that the pre‐hospitalisation phase was an incredibly distressing time. For theme 3: Participants' accounts of hospitalisation framed it overwhelmingly as an appropriate intervention that brought relief and respite. For theme 4: Feeling let down by inadequate service provision was the most starkly recorded and pervasive aspect of parents' experiences of hospitalisation. Predominantly, this was characterised by negative perceptions of services and by feeling disregarded and under‐valued. For theme 5: Participants perceived services and hospitalisation as stigmatising, but also suggested that society at large had negative attitudes towards mental health. The nature and practices of services were experienced as stigmatising and embarrassing	Parents in this study found hospitalisation for early psychosis both containing and a source of relief primarily because they felt their child was safe and accessing treatment. Additionally though, parents found hospitalisation confusing, excluding and stigmatising
Jankovic et al. (2011), England	Of the experiential impact of hospitalisation on the parents of young people with early	31 caregivers 12 male and 19 female‐ 16 were parents; 7 were partners; 4 siblings; 2 children; 1 grandmother; and 1 elderly relative	Not inlcuded	Grounded theory	Themes: 1 relief and conflicting emotions in response to the relative's admission—Relief was a predominant emotion as a response to the relative's admission and it was accompanied byfeelings of guilt and worry; 2 frustration with a delay in getting help—Family caregivers frequently experienced difficulties in obtaining help from services prior to involuntary admission and some thought that services responded to crises rather than prevented them; 3 being given the burden of care by services—Family caregivers experienced increased burden when services shifted the responsibility of caring for their mentally unwell relatives to them.; and 4 difficulties with confidentiality. Confidentiality was a delicate issue with family caregivers wanting more information and a say in decisions when they were responsible for aftercare, and being concerned about confidentiality of information they provided to services	Compulsory admission of a close relative can be a complex and stressful experience for family caregivers. In order for caregivers to be effective partners in care, a balance needs to be struck between valuing their involvement in providing care for a patient and not overburdening them
McCann et al. (1996), England	psychosis	17 relative/friends: 14 relatives were mothers and fathers, brothers and one sister, and three were friends (ages not included)	Not included	The initial phase of this study involved the development of the RAISSE (relative assessment interview, schizophrenia in a secure environment) a specific, semistructured interview (McKeown and McCann 1995) p. 348	Six themes were noted: (1) Life event stress—families described stress surround court case and media, and admission to a forensic hosptial; (2) Continual stress—discussed difficulty talking about the criminal offence with their family member, (3) Self coping‐ strategies—discussed the use of supports and adaptive coping strategies to manage stress; (4) Sings of illness—family members discuss the changes that they noticed prior to the admission of their loved one; (5) Self‐coping maladaptive—desribed suppressing feelings, withdrawing from others due to the offence, and a hostile reactions to stressors. Major contributions of this article suitable for our research prurpose is the Clinical Implication Section	The data have provided an illuminating insight mto the thoughts and feelings of relatives and indicate many ways m which a service for relatives could he tailored It is apparent that many relatives are currently not heing supported, involved or allowed to contrihute to the care of their family memher at Ashworth Hospital
Fernandes Moll et al. (2018), Brazil	To investigate perceptions and expectations of the family member/caregiver regading nursing care provided to psychiatric inpatients at a general hospital	Ten relatives of psychiatric inpatients participated in the study—self appointed as either primary or secondary caregiver	Not included‐descriptive‐exploratory qualitative design	Qualitative—semistructured interviews	Participants expressed overall satisfaction with the quality of nursing care and the communication with family	When family members are allowed to be present during treatment and hospitalisation positive nursing care and engagemtn is experienced
Rose (1985), Vancouver, Canada	To elicit the families' perspectives of their experiences of mental illness and its treatment	Ten family members of seven patients particiated in the study: wives (2), parents (3), siblings (1), daughters and sons (3), and grandmother (1). Ages ranged from 20 to 70 and could broadly be described as lower‐ to upper‐middle class	The perspective of phemenology as developed by Shultz (1962). Involved an inductive and interpretive approach to data analysis	Families of adult psychiatric patients whose duration of hospitalisation was likely to be for to 6 weeks during the time alloted for the study, were asked to participate. Two open‐ended/unstructured interviews were conducted with families during the hospitalisation period. The inital interview (within the first 2 weeks of the patient's hospitalisation) was guided by the review of the literature, the researcher's personal experience, and the work of other researchers. The purpose of the intiial interview was to discuss events leading up to and including hospital admission. Words and phrases that might have different meanings or for which there was a danger of assuming shared meaning, were clarified with the families in subsequent interviews	Families were involved in various activities: (1) learning to identify and adjust to necessary changes in their relationship to the ill relative, particularly when visiting the patient in hospital; (2) identifying the role of various staff in the setting; (3) using informal methods of gathering information about the hospital routine, philosophies, etc.; and (4) continually evaluating the reasons and methods concerning treatment as it applied to their relative	Lack of ligitimate role and poor understnading of treatment contributes to feelings of isloation and hampers their ability to support the patient. Have feelings of alientation dfrom the hospital. Families want to be part of the solution
Rose (1998), Balitmore US	To elicit family caregivers' evaluations of family‐focused interventions as well as informal interactions with health professionals and the relevance of these contaacts to families' needs. Second aim was to identifyfrom the families' perspectives, critical dimensions of future interventions by health professionals that they felt would meet their needs	Six parents, two spouses, one sibling—all participants were white, middle class age range 34–65 years	Not identified	Qualitative descriptive‐exploratory design‐focus groups	Analysis led to four categories: (1) family experiences with the ill relative (2) Perceptions of supportive behaviours (3) Perceptions of non supportive behaviours and, (4) Family coping strategies	
Rose et al. (2004), US	To identify barriers to family care in psychiatric settings and to describe family and provider perspectives about what consitutes effective family care	78 persons participated in 11 groups—2 general family groups (urban and suburban) 30 family members	Not identified	Exploratory qualitative design	Health professionals do not care enough to try; families and hp. in conflict; who neds care the most; connecting with communities	Families need help in managing disturbing behaviours and families desire nonjudgemental communication with health professionals
Sanz‐Osorio et al. (2023), Spain	To analyse the concept, values, and strategic initiatives of humanised care in acute psychiatric units from the perspectives of persons with mental health problems, carers, and professionals	20 participantswith mental health problems (7 women and 13 men; different dx—schizophrenia, bipolar, adjustment disorder, BPD, and schizoaffectve disorder). 20 carer participants 16 women and 4 men mother, daughters, wives, father and husband	Jean Watson—Philosophy and science of Caring	Qualitative grounded theory—foucs group technique	Two categories emerged from the analysis: (1) the meaning that humanised care has for participants‐also identifying basic concepts; (2) the strategic initiatives and proposals to improve mental healthcare	Humanised care are the linkages between values and strategic initiatives
Sari and Duman (2020), Turkey	To reveal experiences of family caregivers of individuals with chronic psychiatric illness	Family caregivers who provided care to 16 individuals with chronic mental illness were interviewed	A descriptive qualitative method was used to define/reveal caregivers' perspectives regarding their caregiving experience. (pg. 39). Data from the interviews were evaluated using qualitative content analysis. In the content analysis, data were processed to obtain concepts and themes	The study was performed between November 2017 and August 2018 at a university hospital adult psychiatry inpatient unit by two researchers (A.S., Z.Ç.D.). Caregivers who met study criteria were interviewed and data were collected. A semistructured interview guide was prepared based on the literature. Interviews lasted for a minimum of 18.36 min and a maximum of 55.05 min. Field notes were made during the interview. The researcher conducting the interviews shared the field notes with the other researcher after each interview	Three themes emerged from the interviews: Illness Management (with subthemes of difficulties regarding illness management, and difficulties regarding the healthcare system), The Caregiver's World: Changes and Effects (with subthemes of changing lives and relationships, and effects of the caregiving process), and Coping From the Caregiver's Point of View	These diffi culties and experiences indicate that psychiatric nurses and other mental health professionals should cooperate with family members by involving them in the treatment and rehabilitation process. Familyoriented programs should be developed according to caregivers' experiences and include content related to stigmatisation, care burden, illness symptoms, and coping with diffi cult behaviour
Sellin et al. (2017), Sweden/Norway	To describe the phenomenon of participation, as experienced by relatives of persons who are subject to inpatient psychiatric care due to a risk of suicide	Eight (Five women and three men, aged between 30 and 80) relatives of patients receiving care from professionals in a psychiatric specialist healthcare context in Sweden	Studied with the guidance of a reflective lifeworld research (RLR) approach, as described by Dahlberg. The methodological principles carried out in this study involve a dialogue between the researchers and the transcribed text	Participants were recruited from the relatives of participants from a previous study (Sellin et al. 2017). Six phenomenon‐oriented interviews were carried out in conversation rooms at a healthcare or a university setting. One participant chose a telephone interview and another participant chose to be interviewed at home. All interviews were conducted by the first author. All participants were encouraged to talk about what mattered from their own perspectives of the phenomenon, with regard to their own subjective experiences	The phenomenon of participation is characterised by “being actively involved in the process in which the person regains the desire to live”. Hence, rather than being related to the delivery of care, participation is about being actively involved in the family member's process of recovery. The meaning of participation is further described by its meaning constituents: (1) struggling for being able to be present for the person at risk of suicide, (2) being able to share everyday life, and (3) nurturing sources for vitality	These insights into the meaning of participation highlight the importance of allowing supportive relatives to be a part of the patient's life, while the person is cared for in an inpatient hospital setting. Thus, participation enables relatives to be acknowledged as resourceful human beings in the patient's recovery process, and thereby facilitates a sense of being able to manage and share life itself together with the person. This means that mental health nurses need to recognise individual variations of relatives' participation processes, and take on the responsibility of acknowledging relatives' lifeworlds
Shor and Shavel (2024), Jerusalem, Israel	To explore the experiences of family members in psychiatric hospitalizations in Israel during the COVID—19 period	75 Adult family members participated: 17 males, 58 females, family relationships = mothers, fathers, children of the hospitalised person and spouses	Not identified	Qualitative study using a survey questionnaire with open‐ended questions	Findings included: (1) Difficulties and frustrations experienced by family members when trying to visit their family member during the psychiatric hospitalisation in COVID; (2) diffciulty trying to maintain communication with professional staff—limited communication and interaction with staff; (3) Lack of help and support services for family members	Visitation restrictions during COVID were more extreme in psychiatric hospitals—in fact many visitations were forbidden. Led to significant disruptiosnwith family members' communication with their ill family member and professional staff—thus prevention the development of meaningful relationships between family members and staff. Also prevented the implimination of family—centred care
Shugar et al. (1992), Toronto Ontario	Study the transfer of psychiatric patients from the point of view of the patients' families, focusing on experiences that families found either satisfactory or unsatisfactory	Thirty‐seven interviews were conducted with 46 family members (17 mothers, 12 fathers, 8 spouses, 8 siblings, and 1 son). (pgs. 303–304)	Not stated, but noted that this study was a part of a larger project that developed a model for assessing and improving inpatient transfers (Shugar et al. 1992). Descriptive answers were compiled, assessed, and classified by the investigators	Family interviews were conducted approximately 30 days after the transfer. Families were interviewed by a social worker who was not involved in the patients' treatment. The 45‐min intereview was organised around a semistructured questionnaire, constructed by the authors, that surveyed the families' experiences before, during, and after the transfer. Included questions about (1) their involvement in planning the transfer, (2) their experiences with the sending and receiving units, (3) the competence and helpfulness of staff, (4) the adequacy of the information they were given, (5) the appropriateness of each setting for their hospitalised relatives, and (6) their recommendations for improving transfers	The most significant issues were involvement of families in planning the transfer, provision of timely and adequate information to families, and availability and support of the staff	Hospitals need to met the families' needs for education and support and for forming constructive partnerships with family members
Stacey et al. (2016), Nottingham, United Kingdom	Examine to what extent decisions about patients' care are perceived to be shared on adult acute mental health wards	Of the 7 focus groups were conducted; there was one group of 6 carers (age and kinship relationship not identified) recruited through a voluntary service user organisation	A critical narrative analysis (CNA) using a framework developed by Langdridge (2007). This form of CNA combines phenomenologically informed narrative methods with a critical theory (Langdridge 2007)	Seven focus groups were conducted with occupational therapists, nurses, service users, carers, psychiatrists, peer‐support workers, and social workers. A separate focus group was conducted for each group of people; therefore, groups fully consisted of people from the same personal or professional group. An interview schedule was used as a guide for each of the focus groups. Each group was facilitated by the same two members of the research team who were mental health professionals within the fields of nursing and psychology. The focus groups lasted between 45 and 90 min	Carers felt no place in decision‐making, and that professionals/system purposefully exclude them. Whereas they saw themselves in the position of knowing the patient best because they were closest to the patient. Noted the power conflict between a parental role and the autonomy of the service user	In conclusion, the discourse of VBP and SDM needs to take account of how differentials of power and the positioning of speakers affect the context in which decisions take place
Vandewalle et al. (2021), Begium	This study aimed to develop an understanding of family members' expectations of care and treatment for their relatives with suicidal ideation	Family members were recruited via adult patients in seven wards‐14 family members (included partners, parents, adult children & siblings	Recovery model	Grounded theory	Analysis revealed one core element and four sub‐elements that captured interpaly between family members' expectations of care & treatment, and their own needs & experiences regarding inpatient services & their relatives' situations. Core element:Struggling to remain hopeful while looking through the lens of uncertainty. Sub‐element 1: Assuming safety as a priority; Sub‐element 2: Looking for a healing approach & environment; sub‐element 3: Counting on continuity of care; sub‐element 4: Wanting to be invovled & supported	Mental health professionals, including nurses, can be more empathetic towards the family members and attuned to their expectations. This can underpin partnerships that help families to deal with their feelings of uncertainty and disempowerment. Such partnerships can flourish in recovery‐oriented mental health services that allow meaningful family involvement
Wilkinson and McAndrew (2008), UK	Aim of the study was to understand, from the perspective of informal carers, what experiences they had when their relative was admitted to an acute psychiatic inpatient setting	4 carer participants (age range 34–56) 2 were mothers, and 2 were spouses	Theoretical model of user involvement Hickey & Kipping (1998)	Hermeneutic phenomenology	Four main themes emerged concerned with carer experience of exclusion from acute psychiatric settings. Theme 1: Powerlessness; Theme 2: Feeling isolated; Theme 3: A need to be recognised & valued; Theme 4: A desire for partnership	The findings reflect the views expressed by carers in other studies, identifying that while carers seek to work in partnership with healthcare professionals, at a clinical level they often feel excluded. The study concludes by discussing ways of improving and promoting carer involvement and advocating a partnership in care approach within acute psychiatry
Wood et al. (2013), location of study town in Northern England	Aims to use the analytical device of the carer's ‘journey’ to explore the extent to which carers seem to be positioned as ‘outsiders’ in the hospital space, the degree to which they experience the hospital space as ‘permeable’ and their individually variable and contingent sense of whether the hospital provides a ‘therapeutic landscape’	9 carers (ages and kinship relationship not identified)	None noted	Social interactionist methodology	Findings: carer experiences: ‘outsiders’ in the inpatient space? Overall findings have been oredered to reflect “the journey” of carers. Starting from home base; Arriving at the hospital; Entering the hospital; Interactions in the ward space; Spaces for family and community living within the hospital	If carers are to be seen as equal partners in the treatment and recovery of mental health service users, then as well as being aesthetically pleasing, safe and secure, it is important that the hospital environment be experienced as ‘permeable’ for them in their caring role
Wood et al. (2021), UK	Aim of this study is to examine the priorities and needs of family and carers of acute psychiatric inpatients who experience psychosis within the context of the COVID19 pandemic. Family and carer experiences were explored from admission through to discharge	14 family/carers four male and ten females, and the average age was 54.29 years	None identified	Exploratory, naturalistic, qualitative study	A total of four themes and 16 subthemes were identified. The four themes were ‘A turbulent journey to hospital admission’, ‘I need information and support’, ‘Maintaining my relationship with my loved one’ and ‘Inpatient care is a mixed bag’ Subthemes for theme 1 are:A struggle to get help Things have to get worse before they get better Distressing psychosis and behaviours Emergency services involvement and sectioning Subthemes for theme 2 are: Limited communication left wanting more Seeking involvement feeling like a burden Carers need support too Subthemes for Theme3 are: Visits and telephone contact are a lifeline but extremely challenging Riding the wave of inpatient care and the impacts on the relationship Offering flexible support and being there Subthemes for Theme 4 are: Medication is the primary treatment Holistic care wanted but not always received The importance of staff continuity and relationships Is my loved one in a safe therapeutic environment? Collaborative discharge planning is essential	The findings demonstrated that family and carers feel excluded from inpatient care and struggled to maintain contact with their loved ones, which was exacerbated by COVID‐19 related restrictions Communication and being regularly informed about their loved one's care, as well as visiting loved ones, was particularly problematic. Inpatient care needs to be more inclusive of family and carers and ensure they are kept in mind at every stage of the admission
Wyder et al. (2018), Brisbane, Australia	Aimed to explore the family's experience of an Involuntary Treatment Order and the impact this had on their caring ability and wellbeing	19 next of kin—12 were female and seven male Fifteen of the participants were parents (six fathers and nine mothers), three were partners, and one was a sibling	None identified	Qualitative interpretivist approach—although the authors appear to describe grounded theory coding methods	The family experiences are closely linked to key events in their relative's illness journey These include: the crisis building; the admission; the time in hospital; discharge from hospital; and the reintegration in the community. Role changes for families were identified as: becoming more involved concerned and worried; moving back and letting go of responsibilty; reconnecting with the person	We suggest four critical elements for providing recovery‐oriented support to families These include: (a) ensuring that families feel that their relative is safe and receiving the care needed; (b) keeping the family informed about their relative's progress; (c) ensuring families have access to information about the mental health system, and (d) working in partnership with the families

### 2.4 Critical Appraisal

Critical appraisal of the included studies was independently completed by three authors (initials to be added after review) using the widely utilised Critical Appraisal Skills Programme (CASP) Qualitative Studies Checklist (2018). The focus of the CASP checklist is on three main components: (a) study validity—two questions on a clear statement of research aim and appropriateness of qualitative methodology and four questions on study design, recruitment, data collection, and the relationship between the researcher and participants, (b) the results—three questions on ethical issues, analysis, and a clear statement of findings, and (c) the value of the research (Critical Appraisal Skills Program 2018). The studies were deemed valuable if the authors contributed to new knowledge, identified areas for new research, and discussed how the research could be used. The assessment of overall value was ascertained using two criteria, useful and limited.

### 2.5 Data Analysis

Descriptive data about the articles (country, publication date, sample and methods) were tabulated. Thematic synthesis of data occurred over three stages following the method of Thomas and Harden (2008). Initially, text findings on families' experiences and perspectives when an adult family member has been hospitalised with a mental illness were coded line by line; followed by the development of descriptive themes (staying close to the primary studies). Lastly, analytical themes were generated to represent a stage of interpretation to generate new interpretive constructs and explanations that can be used as a basis for evidence‐based practice.

## 3 Results

See Figure 1.

**FIGURE 1:**
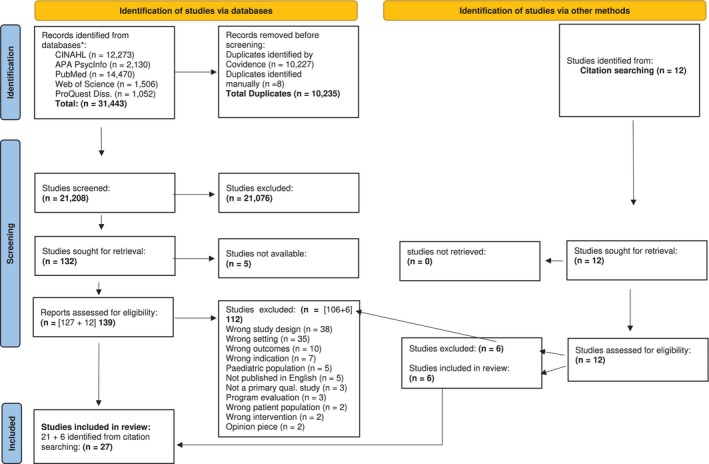
PRISMA flow diagram. *Consider, if feasible to do so, reporting the number of records identified from each database or register searched (rather than the total number across all databases/registers)—Done. **If automation tools were used, indicate how many records were excluded by a human and how many were excluded by automation tools—Done. From: Page et al. (2021).

### 3.1 Included Studies

As shown in Figure 1 (Page et al. 2021), 31 443 studies were identified across the databases. The studies were imported into Covidence, where 10 235 duplicates were removed. Following duplicate removal, 21 208 remained to be screened. Titles/abstracts were initially screened based on the inclusion/exclusion criteria, which excluded 21 076 studies. Three authors (initial to be added following review) independently performed the title and abstract screening process. Any disagreements were jointly reviewed and discussed. Of the 132 studies that were included for full text screening, five studies were not available. The remaining 127 (+12 from citation searching) studies were full text screened, which further excluded 112 studies. The final review dataset was 27 articles.

Publication dates of included articles ranged from 1985 to 2024. Seven of the articles (27%) were published in the last 5 years. Over 44% (*n* = 12) of the articles were published between 2010 and 2018. Most articles originated in the United Kingdom (*n* = 11, 40.7%), followed by Canada (*n* = 4, 14.8%), Australia (*n* = 2, 7.4%), and the United States of America (*n* = 2, 7.4%). The remainder of the articles were from Belgium (*n* = 1, 3.7%), Brazil (*n* = 2, 7.4%), Israel (*n* = 1, 3.7%), Norway (*n* = 1, 3.7%), Spain (*n* = 1 3.7%), Sweden (*n* = 1, 3.7%) and Turkey (*n* = 1, 3.7%). The sample varied across multiple participant groups including families (also referred to as next of kin) (*n* = 16, 59.3%), parents (*n* = 6, 22.2%), carers, professionals, and service users (*n* = 4, 14.8%), and carers (identified as informal and or friends) (*n* = 1, 3.7%). The number of family member/carer participants in each study ranged from three (Crisanti 2000) to 75 (Shor and Shavel 2024). Regarding theoretical frameworks, 13 articles did not identify any theoretical framework. The theoretical frameworks that were identified included: critical realism, Jean Watson philosophy and science of caring, critical theory, model of user involvement, life world research, and the recovery model. Qualitative methodology approaches most often utilised were descriptive, phenomenology, interpretive phenomenology, and critical narrative analysis. Of the 27 included articles, data collection involved interviews (*n* = 17, 63.0%), focus groups (*n* = 5, 18.5%), a combination of interviews and focus groups (*n* = 4, 14.8%), and an open‐ended questionnaire (*n* = 1, 3.7%). Full details of the included studies are provided in Table 3.

### 3.2 Results of Critical Appraisal of Studies

When the results of each reviewer were combined, there was strong agreement across each question in the CASP checklist. Discussion occurred where there were any discrepancies until a consensus was reached. Consistent with the CASP checklist (Critical Appraisal Skills Program 2018), no scores were attributed to articles and no studies were excluded based on study quality. All articles included in this review had a clear statement of the aims of the research and the use of a qualitative methodology was appropriate for our review question focused on human experiences and perspectives. Out of 27 included articles, 22 authors justified the research design and provided some discussion on why the methods were used. In four studies (Buriola et al. 2021; Crisanti 2000; Førde et al. 2016; Shor and Shavel 2024), little detail was provided beyond it being a qualitative study.

In all articles, recruitment was detailed, with participants aligning with our inclusion criteria. Data collection were described in all articles, but detail was often scant on the justification of methods and data saturation. Authors of just over half (51.85% *n* = 14) of the included articles examined their roles, potential bias, and influence on the study, but critical examination of their influence on study design, data collection, and analysis was rare. In the remaining 13 studies, there was no consideration of the researcher role. Across the dataset, detail on ethical issues was appropriate in 74.07% of the studies reviewed (*n* = 20). In the remaining nine studies, ethical issues were not detailed.

Overall, the analysis and presentation of findings were clear in 25 studies. The remainder lacked detail on the analytical process (Crisanti 2000) and lacked a clear statement of findings (McCann et al. 1996). Overall, the majority of authors described how their research contributed to practice or policy and identified new areas for research. There were some good examples where implications for practice and/or research were clearly described (Acres et al. 2022; Allan 2013; Clarke and Winsor 2010; Finlay‐Carruthers et al. 2018; Giacco et al. 2017; Hickman et al. 2016; Rose 1985; Sellin et al. 2017; Sanz‐Osorio et al. 2023; Shugar et al. 1992; Stacey et al. 2016; Vandewalle et al. 2021; Wilkinson and McAndrew 2008; Wood et al. 2021; Wyder et al. 2018).

Full details of the critical appraisal are in Table 4.

**TABLE 4:** Critical appraisal.

	Aims	Methodology	Design	Recruitment	Data	Participants	Ethical issues	Analysis	Findings	Overall value
Arces et al. (Acres et al. 2022)	Yes	Yes	Yes	Yes	Yes	No	Yes	Yes	Yes	Useful
Allan (2013)	Yes	Yes	Yes	Yes	Yes	Yes	Yes	Yes	Yes	Useful
Askey et al. (2009)	Yes	Yes	Yes	Yes	Yes	No	Yes	Yes	Yes	Useful
Buriola et al. (2021)	Yes	Yes	No	Yes	Yes	No	Yes	Yes	Yes	Useful
Clarke and Winsor (2010)	Yes	Yes	Yes	Yes	Yes	No	No	Yes	Yes	Useful
Crisanti (2000)	Yes	Yes	No	Yes	Yes	No	No	No	Yes	Limited
Finlay‐Carruthers et al. (2018)	Yes	Yes	Yes	Yes	Yes	Yes	Yes	Yes	Yes	Useful
Førde et al. (2016)	Yes	No	No	Yes	Yes	No	Yes	Yes	Yes	Useful
Giacco et al. (2017)	Yes	Yes	Yes	Yes	Yes	Yes	Yes	Yes	Yes	Useful
Hickman et al. (2016)	Yes	Yes	Yes	Yes	Yes	Yes	Yes	Yes	Yes	Useful
Jankovic et al. (2011)	Yes	Yes	Yes	Yes	Yes	Yes	Yes	Yes	Yes	Useful
McCann et al. (1996)	Yes	Yes	Yes	Yes	Yes	No	No	Yes	No	Limited
Fernandes Moll et al. (2018)	Yes	Yes	Yes	No	Yes	Yes	Yes	Yes	Yes	Useful
Rose (1985)	Yes	Yes	Yes	Yes	Yes	Yes	No	Yes	Yes	Useful
Rose (1998)	Yes	Yes	Yes	No	Yes	No	No	Yes	Yes	Useful
Rose et al. (2004)	Yes	Yes	Yes	Yes	Yes	No	No	Yes	Yes	Useful
Sanz‐Osorio et al. (2023)	Yes	Yes	Yes	Yes	Yes	No	Yes	Yes	Yes	Useful
Sari and Duman (2020)	Yes	Yes	Yes	Yes	Yes	No	Yes	Yes	Yes	Useful
Sellin et al. (2017)	Yes	Yes	Yes	Yes	Yes	Yes	Yes	Yes	Yes	Useful
Shor and Shavel (2024)	Yes	No	No	Yes	Yes	No	Yes	Yes	Yes	Useful
Shugar et al. 1992)	Yes	Yes	Yes	Yes	Yes	Yes	No	Yes	Yes	Useful
Stacey et al. (2016)	Yes	Yes	Yes	Yes	Yes	Yes	Yes	Yes	Yes	Useful
Vandewalle et al. (2021)	Yes	Yes	Yes	Yes	Yes	Yes	Yes	Yes	Yes	Useful
Wilkinson and McAndrew (2008)	Yes	Yes	Yes	Yes	Yes	Yes	Yes	Yes	Yes	Useful
Wood et al. (2013)	Yes	Yes	Yes	Yes	Yes	Yes	Yes	Yes	Yes	Useful
Wood et al. (2021)	Yes	Yes	Yes	Yes	Yes	Yes	Yes	Yes	Yes	Useful
Wyder et al. (2018)	Yes	Yes	Yes	Yes	Yes	Yes	Yes	Yes	Yes	Useful

### 3.3 Findings of Thematic Synthesis

Three analytical themes were generated by researchers from their analysis: (1) impenetrable system—‘*Expert‐itis’*; (2) perpetuating family invisibility; and (3) unlocking the door.

### 3.4 Impenetrable System—‘*Expert‐Itis’*

The theme of Impenetrable System—‘*Expert‐itis’* refers to participants' perceptions of how the mental health system continues to marginalise, undervalue, and treat family members as outsiders (Allan 2013; Hickman et al. 2016; Stacey et al. 2016). Those within the system often assume the position of expert to monopolise knowledge and control of communication and treatment practices (Allan 2013; Finlay‐Carruthers et al. 2018; Jankovic et al. 2011). This makes the system impenetrable to families and fails to acknowledge and value the personal knowledge and experiences of family members (Allan 2013; Finlay‐Carruthers et al. 2018; Jankovic et al. 2011). Authors reported that participants spoke about having extensive lifelong knowledge about their family members and once they were admitted, the hospital mental health system and staff assumed expertise holding a “monopoly on the truth…” (Allan 2013, 94). In the articles reviewed, participants stated they did not feel a part of the professional team as “professionals did not value or validate their personal knowledge” (Finlay‐Carruthers et al. 2018, 1540), with parental family members reporting they felt they were expected to relinquish their roles as parents while their young adult child was hospitalised (Clarke and Winsor 2010):

We are like outsiders, you're not kept in the loop…you're not involved in anything, you're not kept really up to date with what is going on. (Finlay‐Carruthers et al. 2018, 1540)

The dehumanising process of excluding the knowledge of family members can begin with an initial emergency department contact. Carers discussed being repeatedly ignored or denied communication and information sharing by emergency room nurses; leaving carers feeling voiceless and excluded despite having considerable knowledge and understanding of current issues (Acres et al. 2022).

In the articles reviewed, study participants' views of an impenetrable system were further entrenched through perceived exclusionary practices engaged in by hospital mental health professional staff; practices related to confidentiality and lack of sharing information. While participants appreciated the sensitive nature of confidentiality, they believed that relevant information should be shared as they were often going back to the role of caregiver (Jankovic et al. 2011). Some participants believed that mental health professionals were hiding behind laws like the personal health information acts as a defence to say why they could not share information (Clarke and Winsor 2010; Førde et al. 2016; Rose et al. 2004). The lack of sharing of diagnostic and treatment information and presenting the information in layperson language was deemed by participants as an exclusionary practice (Giacco et al. 2017; Hickman et al. 2016; Rose 1985; Sari and Duman 2020):

You phone up to ask how's your son and they can't give you any information at all. They were very limited. They'd say ‘he's just the same, he's the same as he was’ you know what I mean? So they wouldn't give hardly anything. I don't think it's on really, is it? (Hickman et al. 2016, 150)

In some of the articles reviewed, the practice of hospital discharge meetings was experienced by participants as “token gestures” of inclusion (Allan 2013, 99). Participants recounted discharge meetings as intimidating because these meetings were often set at the last minute with no prior family involvement or notification, and family views when expressed were often ignored or invalidated (Askey et al. 2009; Clarke and Winsor 2010; Wood et al. 2021).

It felt like, this is our [the healthcare teams'] plan. Our plan was to discharge him and, like it wasn't a joint decision. It was like, he's going to get discharged. What do we need to talk about before this happens? Like it was a place to air concerns, but it wasn't a place that decisions were going to get changed or anything you know. At that point I was relatively happy to have him in hospital…relatively concerned about him coming home. (Clarke and Winsor 2010, 245)

While racism can be a factor in the further marginalisation of families; African American families described very similar negative perspectives and experiences (Rose et al. 2004). Families' experiences of not feeling included in the provision of care were compounded by the COVID‐19 pandemic (Shor and Shavel 2024; Wood et al. 2021).

### 3.5 Perpetuating Family Invisibility

Intertwined with study participant views of the impenetrability and ‘expert‐it is’ of the hospital mental health system, participants in all the reviewed articles encountered layers of inhumane attitudes and interactions towards family members from hospital mental health staff (Førde et al. 2016). For family members, these encounters contributed significantly to their own negative emotional states (Allan 2013; Crisanti 2000; Finlay‐Carruthers et al. 2018; Hickman et al. 2016; Shor and Shavel 2024; Shugar et al. 1992; Vandewalle et al. 2021; Wilkinson and McAndrew 2008). Participants believed that their treatment culminated in experiences of isolation and alienation and increased depression and anxiety, giving rise to an awareness of their invisibility to mental health staff (Clarke and Winsor 2010; Shor and Shavel 2024).

Across several articles, participants described strained relationships with unit staff and instead of feeling acknowledged, listened to, supported, and informed, they were met with incivility, hostility, and judgement (Acres et al. 2022; Crisanti 2000; Finlay‐Carruthers et al. 2018; Førde et al. 2016):

Many health professionals did not engage in the most basic courtesies such as returning phone calls or acknowledging the presence of families in hospital corridors. (Rose et al. 2004, 44)

I was considered a nuisance both literally and figuratively. (Stacey et al. 2016, 38)

Some professionals acted as if they did not care and were frequently critical of them [family members]. (Askey et al. 2009, 324)

I believe that the biggest problem is the attitudes towards the patients' families…that they regard you almost as the cause of your son's problems… (Førde et al. 2016, 4)

You don't know me so don't make judgments about me or my family unless you take the time to get to know me. (Rose 1998, 145)

Struggling for engagement and involvement left many participants feeling ridiculed, insecure, frustrated, guilty, helpless, hopeless, demeaned, and powerless (Allan 2013; Clarke and Winsor 2010; Crisanti 2000; Finlay‐Carruthers et al. 2018; Førde et al. 2016; Vandewalle et al. 2021):

Sometimes the professionals don't listen and understand what's actually happening with X. They should listen to what carers are saying more. It makes me feel frustrated. (Askey et al. 2009)

You feel a sense of not being heard, not valued as a human being. You're desperate for help… (Wyder et al. 2018, 326)

### 3.6 Unlocking the Door

This theme highlights family participants' needs when engaging and interacting with the psychiatric hospital care team and identifies what family members perceive influences (facilitates or prevents) their engagement and interactions with the psychiatric hospital care team. The experiences of family members of psychiatric hospital care team member(s) unlocking the door and collaborating with them in the care and treatment of their loved ones are acknowledged.

In several studies, authors reported that participants stated they needed a hospital mental health system and staff that are permeable, open to involvement, and committed to building good relationships with families, sharing information, and collaborating with treatment and discharge planning (Giacco et al. 2017; Wood et al. 2013). While respecting the need for confidentiality, participants believed more could be done to foster information sharing and collaborative partnerships with the healthcare team (Finlay‐Carruthers et al. 2018).

Authors also shared that participants expressed they need the treating team to recognise the critical role they play in the life of their relative and to be respected as experts about their loved one (Giacco et al. 2017). Families stated they needed a supportive and caring approach with nonjudgmental communication from professional staff members:

I was actually asked how I was by the staff on the ward and that was a new experience, really positive thing. Generally concerned and interested which was lovely. (Giacco et al. 2017, 6)

The physical hospital ward environment and space were perceived by study participants in one study to be a barrier to inclusion (Wood et al. 2013). Permanently locked unit doors, nursing staff as gatekeepers to who enters and exits, and privacy limitations were viewed as the controlling hospital institution. A hospital unit that fostered a more ‘homely’ space was deemed to be more positive and inclusionary (Wood et al. 2013).

Family members described supportive behaviours from nursing staff that facilitated cooperation and collaboration. Examples of supportive behaviours that were meaningful to family members were: (1) meeting the primary nurse who was the main contact person; (2) meeting consumers who spoke about living with a mental illness; (3) being provided with concise medication explanations; and (4) sharing their experiences of behaviours they have seen (Rose 1998; Sellin et al. 2017). Behaviours of mental health professionals that were experienced as “caring” were viewed as humanising (Sanz‐Osorio et al. 2023).

When the hospital mental health system opens the door to having family members participate with health professionals in planning for comprehensive hospital care, participants' experiences were predominantly positive (Buriola et al. 2021; Fernandes Moll et al. 2018):

I've always been treated well here. When he is interned, they call me at the meeting, and let me know about the things that happened at the hospital, about his medications, about how he is, about exams, these things, so I feel like helping him to recover. (Buriola et al. 2021, 5)

## 4 Discussion

The three analytical themes: (1) Impenetrable System—‘*Expert‐itis’*; (2) Perpetuating Family Invisibility; and (3) Unlocking the Door were developed from 27 papers spanning over 38 years from several countries. The findings are noteworthy as authors consistently documented multiple family participants' experiences of inhumane professional attitudes and practices in hospital mental health systems. Overall, findings from the reviewed articles demonstrated a consensus towards supporting more family collaboration with psychiatric hospital professionals. These findings align with Doody et al. (2017) who indicated collaborative decisionmaking was thwarted by lack of communication, uncoordinated care planning, constraints blamed on confidentiality, and claims of insider knowledge These findings continue the arguments expressed by other authors (Fleming et al. 2012; Levack et al. 2009; Miller 2012; Abou Seif et al. 2022; Stejskal 2012) that families of people with mental illness want to be involved in all aspects of care; however, the health system continues to restrict their involvement in the planning, delivery, and evaluation of care. While our systematic review focused on acute psychiatric inpatient care, families, and their experiences, authors of an earlier study (Goodwin and Happell 2006) found family involvement is not uniformly positive or in the best interests of the service user. In their qualitative study, Goodwin and Happell (2006) interviewed psychiatric nurses, consumers, and carers and found that the needs and interests of consumers and carers can be mutually exclusive, and it is hard for nurses to navigate priorities of needs and interests.

While navigating family needs and interests might be complex, our findings spotlight the long‐term entrenched negative patterns practised by hospital mental health systems and staff. This raises the question of how mental health hospital systems and practitioners can adopt different attitudes and practices to become more inclusive, more family‐centred, more humane, and more recovery‐focused.

The importance of engaging families in mental healthcare has been recognised internationally, and endorsed by governments and healthcare organisations (Cameron et al. 2022). The World Health Organization's Mental Health Action Plan (2013–2030) champions stakeholder collaboration to motivate and engage stakeholders from all relevant sectors, including people with mental disorders, carers, and family members, in the development and implementation of policies, laws, and services relating to mental health, through a formalised structure and/or mechanism (The World Health Organization 2021). The Australian Government developed principles for partnership and collaboration to ensure all aspects of service design and delivery are co‐designed and addressed, including tools for modelling, needs analysis, implementation, evaluation, and, where necessary, capacity building (Vision 2030 For Mental Health and Suicide Prevention in Australia). A Canadian Provincial Government recommended, as part of their provincial Personal Health Information Act Statutory Review, that consideration be given to amending the legislation to ensure family members and caregivers providing support to, and often living with, an individual with a mental illness or addiction, have access to the appropriate personal health information necessary to provide that support (Government of Newfoundland and Labrador 2022). Despite government policy and recommendations in some countries, there appears to be a disconnect between policy intent and practice as research has revealed the difficulties of implementing family involvement in mental healthcare.

In their systematic review, Eassom et al. (2014) assessed how the involvement of family was implemented in the treatment of people with psychosis. They included 43 articles in their review, focusing solely on staff perspectives. Forty‐two studies featured qualitative data, and 23 studies were completed in the United Kingdom. Their findings suggest that to implement family involvement in care, ALL members of a clinical team should be trained and regularly supervised; thus, utilising a ‘whole team approach’ (Eassom et al. 2014). Alternately, at the practice level it has been recognised that caring for a person with a mental illness requires a significant commitment from families, and family involvement in care planning can be fraught with conflicting experiences related to different requirements between mental health professionals, families, and service users (Doody et al. 2017).

To gain an understanding of family‐centred practice, Foster et al. (2016) completed an integrative review examining published peer‐reviewed literature between 1994 and 2014, focusing on family‐centred practices for children or adults within mental health settings. They noted that while family‐centred practice is reasonably developed in services such as paediatrics, it has not been rigorously explored across adult mental health systems. They found family‐centred practice starts with stakeholders (family allies, clinicians, employers, professional organisations) and a belief that families play a pivotal role in service user recovery and that relationships between health service providers, consumers, and families are key to enabling a whole family approach. However, to cultivate and maintain this approach requires a significant investment in workforce education programs, financial resources, and strong leadership skills (Foster et al. 2016). More recently, according to Carbonell et al. (2020) one major impediment to the implementation of family‐centred care is the lack of support from public healthcare systems and the dominance of biomedical and pharmacological models.

Findings from our review extend and further challenge governments, policymakers, mental healthcare systems, and professionals to collaborate more effectively to promote family involvement in patient care. There is a need for more extensive research on the types of interventions and communication strategies to promote family engagement, and to incorporate both consumers and family members/carers in the design process.

### 4.1 Limitations

A systematic approach to the literature search and the researchers' appraisal of studies using the Critical Appraisal Skills Programme (CASP) and Qualitative Studies Checklist (2018) strengthened this review. However, this review is not without limitations. The English‐only search strategy and the focus on scholarly peer‐reviewed publications and graduate theses may have been limiting factors. In four studies, authors reported on multiple participant groups such as family carers, service users, and service providers. The research team focused analysis on the carer‐specific findings; however, additional detail may have been missed given the varied samples. In this review, we only capture experiences that are part of formal research studies. Studies conducted in different contexts might produce different results; however, the trends identified in our review were consistent. While the goal of qualitative research is not to apply findings directly to other settings and contexts, qualitative findings might be transferable to other contexts (Ravitch 2021). For this systematic qualitative review, the transferability of the findings across all settings, including community services, must be further investigated.

## 5 Conclusions

In this qualitative systematic review, we document families' experiences of the psychiatric hospitalisation of their adult member as broadly unfavourable. To shift the pervasive negative attitudes and behaviours of mental health professionals in hospital settings towards a more collaborative approach with families will first require a seismic philosophical shift from a biomedical unidirectional model of care towards health models incorporating holism and collaboration. A paradigm transformation of how health is viewed and practiced can inform the beliefs, values, and attitudes of governments, policymakers, hospital administrators, program managers, and direct care mental health professionals.

## 6 Relevance to Clinical Practice

As nurses are often at the centre of the treatment of people who are hospitalised in psychiatric settings and are the conduit between the person's family members, it is important that nurses acknowledge family members, both as humans and experts, and make greater attempts to support them and involve them in the care of their loved one. In acute hospital settings, nurses can be more proactive in providing family members with mental health psychoeducation and involving them in care planning, including discharges. These practices would go a long way in fostering a sense of cooperation and collaboration—increasing family visibility. Further research is required to build the evidence that provides best practice for family‐centred care and collaboration within psychiatric hospital settings.

## Author Contributions

**Jane Karpa:** conceptualisation, methodology validation, formal analysis, writing – original draft preparation, visualisation, supervision, project administration, funding acquisition. **Amanda Kenny:** validation, investigation, resources, data curation, writing – original draft preparation; writing – review and editing, supervision. **Andrea Thomson:** validation, investigation, writing – original draft, writing – review and editing. **Marian Ramage:** validation, investigation, resources, data curation, writing – original draft. **Monica Ginn Forsyth:** investigation, writing – review and editing. **Kendra Rieger:** writing – review and editing.

## Ethics Statement

The authors have nothing to report.

## Conflicts of Interest

The authors declare no conflicts of interest.

## Supporting information


**Data S1.** PRESS review materials.

**Data S2.** PRESS review materials.

**Data S3.** Searches used in data bases.

## Data Availability

The data that support the findings of this study are available from the corresponding author upon reasonable request.
