# Supplementary material for: Experiences and Perspectives of Families of Psychiatric Hospitalisation of Their Adult Family Member: A Qualitative Systematic Review
Source: Int J Ment Health Nurs. 2025 Jul 9;34(4):e70042. doi: 10.1111/inm.70042 (PMC12241760; doi:10.1111/inm.70042)
Supplement: Supplementary file 1 — Data S1. PRESS review materials. [file INM-34-0-s003.docx]

**PRESS Guideline – Search Submission & Peer Review Assessment Revisions and Notes – April 2, 2022**

**1. Translation**

No revisions

**2. Boolean and proximity Operators**

No revisions

**3. Subject Headings**

No revisions

**4. Text word searching**

No revisions

**5. Spelling, syntax, and line numbers**

Overall Required Revision: For correct results on TI, AB, and MM lines (e.g. to ensure that the database searches each term/phrase in the correct field): give TI, AB, or MM at the beginning of the line, then enclose the entire search string that follows in brackets

S1 – Required Revision: begins TI TI. Reduce to one TI

S2 – Required Revision: begins AB AB. Reduce to one AB

S3 – Required Revision: remove brackets from around the subject headings (but leave the quotation marks)

S5 and S6 – Required Revision: (emergency AND ((W0 room*) OR (W0 department*)) returns 0 results.

Change to emergency W0 room* OR emergency W0 department*

S5 and S6 – Required Revision: (acute AND (W0 care) OR (W0 inpatient*) returns 0 results.

Change to acute W0 care OR acute W0 inpatient*

S13 and 14 – Required Revision: Typo – family W**O** cent#red W0 care: Change O to 0

S13 – Not a Required or Suggested Revision:

A note for W0 proximity phrases with no truncation: when not adding truncation, phrases can be more simply done as phrase searching using quotation marks. For example, shared W0 decision W0 making could also be accomplished with “shared decision making”. A comparison of the two shows a difference of 1 result, where the additional hit included the phrase “shared decision making’s”. Of note is that changing the phrase to be shared W0 decision W0 making* (or “shared decision making*”) includes additional results entirely composed of “shared decision making” followed by an ellipsis

**6. Limits and filters**

Not a Required or suggested revision:

A note for efficiency: you can apply the English language filter to the final search line rather than to each individual line to save time while searching, and to easily remove this limiter if your research group decides to re-run the search including results written in other languages.

**7. Overall evaluation**

You will notice that the revisions suggested above will have a significant effect on the number of results that the search retrieves. As submitted, your final number of results on S17 is 1,368. With the revisions suggested, your final number of results will be (or be in excess of, if run after April 2, 2022) 4,598.

Suggested Revised CINAHL Search Strategy with Re-run Results (April 2, 2022)

| # | Query | Limiters/Expanders | Last Run Via | Results |
| --- | --- | --- | --- | --- |
| S17 | S4 AND S8 AND S12 AND S16 | Limiters - English Language  Search modes - Boolean/Phrase | Interface - EBSCOhost Research Databases  Search Screen - Advanced Search  Database - CINAHL | 4,598 |
| S16 | S13 OR S14 OR S15 | Limiters - English Language  Search modes - Boolean/Phrase | Interface - EBSCOhost Research Databases  Search Screen - Advanced Search  Database - CINAHL | 2,337,466 |
| S15 | MM ("Life experiences" OR "decision making, family" OR "dissent and disputes" OR "Family centered care" OR "professional-family relations" OR "Support, Psychosocial") | Limiters - English Language  Search modes - Boolean/Phrase | Interface - EBSCOhost Research Databases  Search Screen - Advanced Search  Database - CINAHL | 58,634 |
| S14 | AB ( treatment* OR intervention* OR need* OR involvement OR Collaborat* OR Perception* OR Perspective* OR Attitude* OR Impression* OR Opinion* OR experience* OR Liv* W0 experience* OR discharge W0 plan* OR Health W0 system W0 responsiveness OR Shared W0 decision W0 making OR family W0 cent#red W0 care OR TI Care W0 plan*) | Limiters - English Language  Search modes - Boolean/Phrase | Interface - EBSCOhost Research Databases  Search Screen - Advanced Search  Database - CINAHL | 1,972,578 |
| S13 | TI ( treatment* OR intervention* OR need* OR involvement OR Collaborat* OR Perception* OR Perspective* OR Attitude* OR Impression* OR Opinion* OR experience* OR Liv* W0 experience* OR discharge W0 plan* OR Health W0 system W0 responsiveness OR Shared W0 decision W0 making OR family W0 cent#red W0 care OR TI Care W0 plan*) | Limiters - English Language  Search modes - Boolean/Phrase | Interface - EBSCOhost Research Databases  Search Screen - Advanced Search  Database - CINAHL | 752,899 |
| S12 | S9 OR S10 OR S11 | Limiters - English Language  Search modes - Boolean/Phrase | Interface - EBSCOhost Research Databases  Search Screen - Advanced Search  Database - CINAHL | 970,690 |
| S11 | MM ("Extended Family" OR Family OR Siblings OR "Significant Other" OR Spouses OR "Visitors to Patients") | Limiters - English Language  Search modes - Boolean/Phrase | Interface - EBSCOhost Research Databases  Search Screen - Advanced Search  Database - CINAHL | 34,212 |
| S10 | AB (Families OR family W0 member* OR Family OR Parent* OR Caregiver* OR Carer* OR Relative* Or Spous* OR Significant W0 other* OR Partner* OR Loved W0 one* OR Stakeholder* OR Mother* OR Sibling* OR Sister* OR Brother* OR Father* OR Wife OR Wives OR Husband* OR Daughter* OR Son OR Sons OR Grandparent* OR Grandmother* OR Grandfather* OR Niece* OR Nephew* OR Cousin* OR Uncle* OR Aunt* OR Proxy OR Proxies OR Friend* OR Boyfriend* OR Girlfriend* OR Elder* OR Adult W0 Child*) | Limiters - English Language  Search modes - Boolean/Phrase | Interface - EBSCOhost Research Databases  Search Screen - Advanced Search  Database - CINAHL | 839,596 |
| S9 | TI (Families OR family W0 member* OR Family OR Parent* OR Caregiver* OR Carer* OR Relative* Or Spous* OR Significant W0 other* OR Partner* OR Loved W0 one* OR Stakeholder* OR Mother* OR Sibling* OR Sister* OR Brother* OR Father* OR Wife OR Wives OR Husband* OR Daughter* OR Son OR Sons OR Grandparent* OR Grandmother* OR Grandfather* OR Niece* OR Nephew* OR Cousin* OR Uncle* OR Aunt* OR Proxy OR Proxies OR Friend* OR Boyfriend* OR Girlfriend* OR Elder* OR Adult W0 Child*) | Limiters - English Language  Search modes - Boolean/Phrase | Interface - EBSCOhost Research Databases  Search Screen - Advanced Search  Database - CINAHL | 296,788 |
| S8 | S5 OR S6 OR S7 | Limiters - English Language  Search modes - Boolean/Phrase | Interface - EBSCOhost Research Databases  Search Screen - Advanced Search  Database - CINAHL | 179,058 |
| S7 | MM ("Hospitals, Psychiatric" OR "Psychiatric Units" OR "Emergency Service" OR "Emergency Services, Psychiatric" OR "Acute Care") | Limiters - English Language  Search modes - Boolean/Phrase | Interface - EBSCOhost Research Databases  Search Screen - Advanced Search  Database - CINAHL | 42,674 |
| S6 | AB (emergency W0 room* OR emergency W0 department* OR health W0 service* OR acute W0 care OR acute W0 inpatient* OR psychiatric W0 hospital*) | Limiters - English Language  Search modes - Boolean/Phrase | Interface - EBSCOhost Research Databases  Search Screen - Advanced Search  Database - CINAHL | 137,443 |
| S5 | TI (emergency W0 room* OR emergency W0 department* OR health W0 service* OR acute W0 care OR acute W0 inpatient* OR psychiatric W0 hospital*) | Limiters - English Language  Search modes - Boolean/Phrase | Interface - EBSCOhost Research Databases  Search Screen - Advanced Search  Database - CINAHL | 53,238 |
| S4 | S1 OR S2 OR S3 | Limiters - English Language  Search modes - Boolean/Phrase | Interface - EBSCOhost Research Databases  Search Screen - Advanced Search  Database - CINAHL | 133,241 |
| S3 | MM ("Psychiatric Patients" OR "Mental Disorders" OR "Mental Disorders, Chronic" OR "Psychiatric Emergencies" OR "Acute Disease" OR "Patient Admission") | Limiters - English Language  Search modes - Boolean/Phrase | Interface - EBSCOhost Research Databases  Search Screen - Advanced Search  Database - CINAHL | 64,181 |
| S2 | AB ( ( mental W0 health AND (condition* OR disorder* OR problem* OR issue*) ) OR "mental illness*" OR "severe mental illness*" OR "mental disorder*" OR "psychiatric disorder*" OR "psychiatric illness*" ) OR ( (psychiatrically OR mentally) AND ill ) ) | Limiters - English Language  Search modes - Boolean/Phrase | Interface - EBSCOhost Research Databases  Search Screen - Advanced Search  Database - CINAHL | 87,275 |
| S1 | TI ( ( mental W0 health AND (condition* OR disorder* OR problem* OR issue*) ) OR "mental illness*" OR "severe mental illness*" OR "mental disorder*" OR "psychiatric disorder*" OR "psychiatric illness*" ) OR ( (psychiatrically OR mentally) AND ill ) ) | Limiters - English Language  Search modes - Boolean/Phrase | Interface - EBSCOhost Research Databases  Search Screen - Advanced Search  Database - CINAHL | 25,937 |
