# Supplementary material for: Experiences and Perspectives of Families of Psychiatric Hospitalisation of Their Adult Family Member: A Qualitative Systematic Review
Source: Int J Ment Health Nurs. 2025 Jul 9;34(4):e70042. doi: 10.1111/inm.70042 (PMC12241760; doi:10.1111/inm.70042)
Supplement: Supplementary file 2 — Data S2. PRESS review materials. [file INM-34-0-s001.docx]

# PRESS Guideline — Search Submission & Peer Review Assessment**

## SEARCH SUBMISSION: THIS SECTION TO BE FILLED IN BY THE SEARCHER

Date submitted: May 23, 2022

### Systematic Review Title:

Experiences and perspectives of families on psychiatric hospitalization of their adult family member: a qualitative systematic review

### This search strategy is …

|  | My PRIMARY (core) database strategy — First time submitting a strategy for search question and database |
| --- | --- |
|  | My PRIMARY (core) strategy — Follow-up review NOT the first time submitting a strategy for search question and database. If this is a response to peer review, itemize the changes made to the review suggestions. |
| X | SECONDARY search strategy— First time submitting a strategy for search question and database |
|  | SECONDARY search strategy — NOT the first time submitting a strategy for search question and database. If  this is a response to peer review, itemize the changes made to the review suggestions |

### Database:

PubMed

### Interface

National Library of Medicine <https://pubmed.ncbi.nlm.nih.gov/>

### Research Question

The purpose of this search is to discover all publications in this database that describe families’ experiences of involvement with an acute care health setting, when their adult family member is hospitalized for psychiatric reasons.

### PICO Format

| **P** | Adult with an acute mental disorder |
| --- | --- |
| **I** | n/a |
| **C** | n/a |
| **O** | n/a |
| **S** | n/a |

The PICO format does not correspond to the concepts of this systematic review. The research concepts are:

Concept 1: Person with an Acute Mental Disorder

Concept 2: Emergency Health Care Setting

Concept 3: Family members of the person with the mental disorder

Concept 4: Experiences of the patient’s family members with the health care staff

### Inclusion Criteria

Adult participants (18 years or older) who are family members or family carers of an adult family member who has been hospitalized with a mental illness.

### Exclusion Criteria

Studies with people under 18 will be excluded.

### Was a search filter applied?

Yes No X

### Other notes or comments you feel would be useful for the peer reviewer?

The following filters were applied to the result lists:

Species – “Humans”

Language – “English”

No age filter was used, as this may not always be applied consistently.

No date filter was applied.

### Please copy and paste your search strategy here, exactly as run, including the number of hits per line.

Contained in separate Excel sheet.

## PEER REVIEW ASSESSMENT: THIS SECTION TO BE FILLED IN BY THE REVIEWER

Reviewer: XXXXXX

Email: XXXXXX

Date completed: May 30, 2022

### TRANSLATION

| A -­‐No revisions | X |
| --- | --- |
| B -­‐ Revision(s) suggested | ☐ |
| C -­‐ Revision(s) required | ☐ |

If “B” or “C,” please provide an explanation or example:

N/A

### 2. BOOLEAN AND PROXIMITY OPERATORS

| A -­‐No revisions | X |
| --- | --- |
| B -­‐ Revision(s) suggested | ☐ |
| C -­‐ Revision(s) required | ☐ |

If “B” or “C,” please provide an explanation or example:

N/A

### 3. SUBJECT HEADINGS

| A -­‐No revisions | ☐ |
| --- | --- |
| B -­‐ Revision(s) suggested | X |
| C -­‐ Revision(s) required | ☐ |

If “B” or “C,” please provide an explanation or example:

- Under Concept 2, consider adding MeSH term (or text word substitutions) for “Mental Health Services”, “Psychiatric Department, Hospital”, “Involuntary Treatment, Psychiatric” and “Psychiatric Nursing”. These expand the contexts in which mental health care takes place, bring in the nursing specialty that deals with mental health, and build on the concept of involuntary mental health care (i.e., treatment and commitment).
- Under Concept 3, consider MeSH term (or text word substitution) for “Legal Guardians” to expand on the concept of proxies/healthcare decision makers.

### 4. TEXT WORD SEARCHING

| A -­‐No revisions | ☐ |
| --- | --- |
| B -­‐ Revision(s) suggested | X |
| C -­‐ Revision(s) required | ☐ |

If “B” or “C,” please provide an explanation or example:

- Under Concept 4, consider adding "health care system response*"[Title/Abstract] and "healthcare system response*"[Title/Abstract] as a text search phrases, in addition to "health system response*"[Title/Abstract], which has already been included in the search terms. That should catch the different uses and spellings of that concept.

### 5. SPELLING, SYNTAX, AND LINE NUMBERS

| A -­‐No revisions | ☐ |
| --- | --- |
| B -­‐ Revision(s) suggested | ☐ |
| C -­‐ Revision(s) required | X |

If “B” or “C,” please provide an explanation or example:

- There are some things going on in the details search detail the final query that are likely an artifact of the PubMed search builder. These should be cleaned up for clarity.
  - The phrase ("humans"[MeSH Terms] AND "English"[Language]) repeats in a few places. I don’t think this impacts the outcomes of the search, but for fussy syntax purposes, the phrase should be removed entirely as it duplicates the action of the filters (See attached search syntax).
  - There are brackets around the MeSH terms in each component of the search. The sample search syntax I attached removed the brackets around the MeSH terms in each section. It didn’t seem to impact the number of results, but it does give the search string clarity.
- Under Concept 4:
- I think there may be a truncation typo in "impressions*"[Title/Abstract]. I think it should be "impression*"[Title/Abstract] instead.
  - Same with "health system respons*"[Title/Abstract]. I think it should be "health system response*"[Title/Abstract].
  - The term "experience*"[Title/Abstract] should also capture "lived experience*"[Title/Abstract]. The sample search syntax I attached removed “lived experience”. It didn’t seem to impact the number of results.

### 6. LIMITS AND FILTERS

| A -­‐No revisions | X |
| --- | --- |
| B -­‐ Revision(s) suggested | ☐ |
| C -­‐ Revision(s) required | ☐ |

If “B” or “C,” please provide an explanation or example:

- It is a good choice not to apply age filters. Even where they are used appropriately, they are too ambiguous to be used meaningfully, particularly in the context of a search with two age group-based selection criteria.

### OVERALL EVALUATION (Note: If one or more “revision required” is noted above, the response below must be “revisions required”.)

| A -­‐No revisions | ☐ |
| --- | --- |
| B -­‐ Revision(s) suggested | ☐ |
| C -­‐ Revision(s) required | X |

### Additional comments:

- Overall, this is a great search strategy. You did a great job of translating the research question into an effective search string! Most of my suggestions relate to a few terminology considerations and some minor syntax changes.
- The way you have mapped core concepts to sections of your search strategy is helpful. It makes the search strings easy to read and interpret. PICO can be challenging to use for systematic reviews of qualitative evidence. The format for PICO changes a little bit in this context. I still find PICO helpful to use as a guideline for not only search strategies, but for researchers when they are evaluating evidence. If you want to use it or suggest it, in this case, it would be:
  - P (characteristics of the Population/Patient): Adult family members/caregivers of adult psychiatric patients.
  - I (event/activity/experience/process of Interest): Family member/caregiver lived experiences with the healthcare system/healthcare staff.
  - Co (Context in which the event/activity/experience/process of Interest takes place): the acute/emergency health care setting when their loved one is hospitalized for mental health reasons.

**Adapted from:

McGowan, J., Sampson, M., Salzwedel, D. M., Cogo, E., Foerster, V., & Lefebvre, C. (2016). PRESS peer review of electronic search strategies: 2015 guideline statement, Appendix A. *Journal of clinical epidemiology*, *75*, 40-46.
