# Supplementary material for: Experiences and Perspectives of Families of Psychiatric Hospitalisation of Their Adult Family Member: A Qualitative Systematic Review
Source: Int J Ment Health Nurs. 2025 Jul 9;34(4):e70042. doi: 10.1111/inm.70042 (PMC12241760; doi:10.1111/inm.70042)
Supplement: Supplementary file 3 — Data S3. Searches used in data bases. [file INM-34-0-s002.docx]

**Supplementary material**

Figure 1. Search strings used in each database.

| **Databases** | **Search** | **# of results** |
| --- | --- | --- |
| CINAHL | TI ( ( mental W0 health AND (condition* OR disorder* OR problem* OR issue*) ) OR "mental illness*" OR "severe mental illness*" OR "mental disorder*" OR "psychiatric disorder*" OR "psychiatric illness*" ) OR ( (psychiatrically OR mentally) AND ill ) ) OR AB ( ( mental W0 health AND (condition* OR disorder* OR problem* OR issue*) ) OR "mental illness*" OR "severe mental illness*" OR "mental disorder*" OR "psychiatric disorder*" OR "psychiatric illness*" ) OR ( (psychiatrically OR mentally) AND ill ) ) OR MM ("Psychiatric Patients" OR "Mental Disorders" OR "Mental Disorders, Chronic" OR "Psychiatric Emergencies" OR "Acute Disease" OR "Patient Admission") **AND** TI (emergency W0 room* OR emergency W0 department* OR (emergency W0 health W0 service*) OR acute W0 care OR acute W0 inpatient* OR psychiatric W0 hospital*) OR AB (emergency W0 room* OR emergency W0 department* OR (emergency W0 health W0 service*) OR acute W0 care OR acute W0 inpatient* OR psychiatric W0 hospital*) OR MM ("Hospitals, Psychiatric" OR "Psychiatric Units" OR "Emergency Service" OR "Emergency Services, Psychiatric" OR "Acute Care") **AND** TI (Families OR family W0 member* OR Family OR Parent* OR Caregiver* OR Carer* OR Relative* Or Spous* OR Significant W0 other* OR Partner* OR Loved W0 one* OR Stakeholder* OR Mother* OR Sibling* OR Sister* OR Brother* OR Father* OR Wife OR Wives OR Husband* OR Daughter* OR Son OR Sons OR Grandparent* OR Grandmother* OR Grandfather* OR Niece* OR Nephew* OR Cousin* OR Uncle* OR Aunt* OR Proxy OR Proxies OR Friend* OR Boyfriend* OR Girlfriend* OR Elder* OR Adult W0 Child*) OR AB (Families OR family W0 member* OR Family OR Parent* OR Caregiver* OR Carer* OR Relative* Or Spous* OR Significant W0 other* OR Partner* OR Loved W0 one* OR Stakeholder* OR Mother* OR Sibling* OR Sister* OR Brother* OR Father* OR Wife OR Wives OR Husband* OR Daughter* OR Son OR Sons OR Grandparent* OR Grandmother* OR Grandfather* OR Niece* OR Nephew* OR Cousin* OR Uncle* OR Aunt* OR Proxy OR Proxies OR Friend* OR Boyfriend* OR Girlfriend* OR Elder* OR Adult W0 Child*) OR MM ("Extended Family" OR Family OR Siblings OR "Significant Other" OR Spouses OR "Visitors to Patients") **AND** TI ( treatment* OR intervention* OR need* OR involvement OR Collaborat* OR Perception* OR Perspective* OR Attitude* OR Impression* OR Opinion* OR experience* OR Liv* W0 experience* OR discharge W0 plan* OR Health W0 system W0 responsiveness OR Shared W0 decision W0 making OR family W0 cent#red W0 care OR TI Care W0 plan*) OR AB ( treatment* OR intervention* OR need* OR involvement OR Collaborat* OR Perception* OR Perspective* OR Attitude* OR Impression* OR Opinion* OR experience* OR Liv* W0 experience* OR discharge W0 plan* OR Health W0 system W0 responsiveness OR Shared W0 decision W0 making OR family W0 cent#red W0 care OR TI Care W0 plan*) OR MM ("Life experiences" OR "decision making, family" OR "dissent and disputes" OR "Family centered care" OR "professional-family relations" OR "Support, Psychosocial") | 4,665 |
| PsycInfo | TI (Families OR family W0 member* OR Family OR Parent* OR Caregiver* OR Carer* OR Relative* Or Spous* OR Significant W0 other* OR Partner* OR Loved W0 one* OR Stakeholder* OR Mother* OR Sibling* OR Sister* OR Brother* OR Father* OR Wife OR Wives OR Husband* OR Daughter* OR Son OR Sons OR Grandparent* OR Grandmother* OR Grandfather* OR Niece* OR Nephew* OR Cousin* OR Uncle* OR Aunt* OR Proxy OR Proxies OR Friend* OR Boyfriend* OR Girlfriend* OR Elder* OR Adult W0 Child*) OR AB (Families OR family W0 member* OR Family OR Parent* OR Caregiver* OR Carer* OR Relative* Or Spous* OR Significant W0 other* OR Partner* OR Loved W0 one* OR Stakeholder* OR Mother* OR Sibling* OR Sister* OR Brother* OR Father* OR Wife OR Wives OR Husband* OR Daughter* OR Son OR Sons OR Grandparent* OR Grandmother* OR Grandfather* OR Niece* OR Nephew* OR Cousin* OR Uncle* OR Aunt* OR Proxy OR Proxies OR Friend* OR Boyfriend* OR Girlfriend* OR Elder* OR Adult W0 Child*) OR MM (Family OR "Family Members" OR "Social Support" OR Parents OR Spouses OR "Significant Others") **AND** TI (emergency W0 room* OR emergency W0 department* OR (emergency W0 health W0 service*) OR acute W0 care OR acute W0 inpatient* OR psychiatric W0 hospital*) OR AB (emergency W0 room* OR emergency W0 department* OR (emergency W0 health W0 service*) OR acute W0 care OR acute W0 inpatient* OR psychiatric W0 hospital*) OR MM ("Emergency Medicine" OR "Crisis Intervention" OR "Psychiatric Hospitals" OR "Psychiatric Hospitalization") AND TI (Families OR family W0 member* OR Family OR Parent* OR Caregiver* OR Carer* OR Relative* Or Spous* OR Significant W0 other* OR Partner* OR Loved W0 one* OR Stakeholder* OR Mother* OR Sibling* OR Sister* OR Brother* OR Father* OR Wife OR Wives OR Husband* OR Daughter* OR Son OR Sons OR Grandparent* OR Grandmother* OR Grandfather* OR Niece* OR Nephew* OR Cousin* OR Uncle* OR Aunt* OR Proxy OR Proxies OR Friend* OR Boyfriend* OR Girlfriend* OR Elder* OR Adult W0 Child*) OR AB (Families OR family W0 member* OR Family OR Parent* OR Caregiver* OR Carer* OR Relative* Or Spous* OR Significant W0 other* OR Partner* OR Loved W0 one* OR Stakeholder* OR Mother* OR Sibling* OR Sister* OR Brother* OR Father* OR Wife OR Wives OR Husband* OR Daughter* OR Son OR Sons OR Grandparent* OR Grandmother* OR Grandfather* OR Niece* OR Nephew* OR Cousin* OR Uncle* OR Aunt* OR Proxy OR Proxies OR Friend* OR Boyfriend* OR Girlfriend* OR Elder* OR Adult W0 Child*) OR MM (Family OR "Family Members" OR "Social Support" OR Parents OR Spouses OR "Significant Others") **AND** TI ( treatment* OR intervention* OR need* OR involvement OR Collaborat* OR Perception* OR Perspective* OR Attitude* OR Impression* OR Opinion* OR experience* OR Liv* W0 experience* OR discharge W0 plan* OR Health W0 system W0 responsiveness OR Shared W0 decision W0 making OR family W0 cent#red W0 care OR TI Care W0 plan*) OR AB ( treatment* OR intervention* OR need* OR involvement OR Collaborat* OR Perception* OR Perspective* OR Attitude* OR Impression* OR Opinion* OR experience* OR Liv* W0 experience* OR discharge W0 plan* OR Health W0 system W0 responsiveness OR Shared W0 decision W0 making OR family W0 cent#red W0 care OR TI Care W0 plan*) OR MM ("Health Personnel Attitudes" OR "Health Care Delivery" OR "Health Care Services" OR "Patient Centered Care" OR "Satisfaction") | 2,024 |
| PubMed | ("mental illness*"[Title/Abstract] OR "severe mental illness*"[Title/Abstract] OR "mental disorder*"[Title/Abstract] OR "psychiatric disorder*"[Title/Abstract] OR "psychiatric illness*"[Title/Abstract] OR "mentally ill"[Title/Abstract] OR "mental health condition*"[Title/Abstract] OR "mental health disorder*"[Title/Abstract] OR "mental health problem*"[Title/Abstract] OR "mental health issue*"[Title/Abstract] OR "psychiatrically ill"[Title/Abstract]) OR ("Mental Disorders"[Mesh] OR "Mentally Ill Persons"[Mesh]) **AND** ("Emergency room*"[Title/Abstract] OR "emergency department*"[Title/Abstract] OR "emergency health service*"[Title/Abstract] OR "psychiatric hospital*"[Title/Abstract] OR "acute care"[Title/Abstract] OR "acute inpatient*"[Title/Abstract]) OR ("Emergency Service, Hospital"[Mesh] OR "Involuntary Commitment"[Mesh] OR "Commitment of Mentally Ill"[Mesh] OR "Emergency Medical Services"[Mesh] OR "Intensive Care Units"[Mesh] OR "Hospitals, Psychiatric"[Mesh] OR "Critical Care"[Mesh] OR "Emergency Services, Psychiatric"[Mesh]) OR ] OR “Mental Health Services”[MeSH Terms] OR “Psychiatric Department, Hospital”[MeSH Terms] OR “Involuntary Treatment, Psychiatric”[MeSH Terms] OR “Psychiatric Nursing”[MeSH Terms] **AND** (Families[Title/Abstract] OR "family member*"[Title/Abstract] OR family[Title/Abstract] OR parent*[Title/Abstract] OR caregiver*[Title/Abstract] OR carer*[Title/Abstract] OR relative*[Title/Abstract] OR spouse*[Title/Abstract] OR "adult child*"[Title/Abstract] OR partner*[Title/Abstract] OR stakeholder*[Title/Abstract] OR mother*[Title/Abstract] OR sibling*[Title/Abstract] OR sister*[Title/Abstract] OR brother*[Title/Abstract] OR father*[Title/Abstract] OR wife[Title/Abstract] OR wives[Title/Abstract] OR husband*[Title/Abstract] OR daughter*[Title/Abstract] OR son[Title/Abstract] OR sons[Title/Abstract] OR grandparent*[Title/Abstract] OR grandmother*[Title/Abstract] OR grandfather*[Title/Abstract] OR proxy[Title/Abstract] OR proxies[Title/Abstract] OR friend*[Title/Abstract] OR boyfriend*[Title/Abstract] OR "significant other*"[Title/Abstract] OR girlfriend*[Title/Abstract] OR elder*[Title/Abstract] OR "loved one*"[Title/Abstract] OR niece*[Title/Abstract] OR nephew*[Title/Abstract] OR cousin*[Title/Abstract] OR uncle*[Title/Abstract] OR aunt*[Title/Abstract]) OR ("Friends"[Mesh] OR "Proxy"[Mesh] OR "Grandparents"[Mesh] OR "Siblings"[Mesh] OR "Spouses"[Mesh] OR "Family"[Mesh] OR "Parents"[Mesh] OR "Adult Children"[Mesh] OR "Caregivers"[Mesh]) OR "Legal Guardians"[Mesh] **AND** (Intervention*[Title/Abstract] OR attitude*[Title/Abstract] OR "discharge plan*"[Title/Abstract] OR "shared decision making"[Title/Abstract] OR "family centered care"[Title/Abstract] OR "care plan*"[Title/Abstract] OR treatment*[Title/Abstract] OR need*[Title/Abstract] OR involvement[Title/Abstract] OR collaboration[Title/Abstract] OR perception*[Title/Abstract] OR perspective*[Title/Abstract] OR impression*[Title/Abstract] OR opinion*[Title/Abstract] OR experience*[Title/Abstract] OR "health system response*"[Title/Abstract]) OR "health care system response*"[Title/Abstract] OR "healthcare system response*"[Title/Abstract] OR ("Psychosocial Intervention"[Mesh] OR "Attitude of Health Personnel"[Mesh] OR "Patient Discharge"[Mesh] OR "Decision Making, Shared"[Mesh] OR "Family Nursing"[Mesh] OR "Patient Discharge Summaries"[Mesh]) | 13,962 |
| Web of Science | "mental illness*" OR "severe mental illness*" OR "mental disorder*" OR "psychiatric disorder*" OR "psychiatric illness*" OR "mentally ill" OR "mental health condition*" OR "mental health disorder*" OR "mental health problem*" OR "mental health issue*" OR "psychiatrically ill" OR "mentally ill person*" **AND** "Emergency room*" OR "emergency department*" OR "emergency health service*" OR "psychiatric hospital*" OR "acute care" OR "acute inpatient*" OR "Emergency Service, Hospital"[Mesh] OR "Involuntary Commitment"[Mesh] OR "Commitment of Mentally Ill"[Mesh] OR "Emergency Medical Services"[Mesh] OR "Intensive Care Units"[Mesh] OR "Hospitals, Psychiatric"[Mesh] OR "Critical Care"[Mesh] OR "Emergency Services, Psychiatric"[Mesh] OR “Mental Health Services”[Mesh] OR “Psychiatric Department, Hospital”[Mesh] OR “Involuntary Treatment, Psychiatric”[Mesh] OR “Psychiatric Nursing”[Mesh] **AND** Families OR "family member*" OR family OR parent* OR caregiver* OR carer* OR relative* OR spouse* OR "adult child*" OR partner* OR stakeholder* OR mother* OR sibling* OR sister* OR brother* OR father* OR wife OR wives OR husband* OR daughter* OR son OR sons OR grandparent* OR grandmother* OR grandfather* OR proxy OR proxies OR friend* OR boyfriend* OR "significant other*" OR girlfriend* OR elder* OR "loved one*" OR niece* OR nephew* OR cousin* OR uncle* OR aunt* OR "Friends"[Mesh] OR "Proxy"[Mesh] OR "Grandparents"[Mesh] OR "Siblings"[Mesh] OR "Spouses"[Mesh] OR "Family"[Mesh] OR "Parents"[Mesh] OR "Adult Children"[Mesh] OR "Caregivers"[Mesh] OR "Legal Guardians"[Mesh] **AND** Intervention* OR attitude* OR "discharge plan*" OR "shared decision making" OR "family centered care" OR "care plan*" OR treatment* OR need* OR involvement OR collaboration OR perception* OR perspective* OR impressions* OR opinion* OR experience* OR "lived experience*" OR "health system respons*" OR "Psychosocial Intervention"[Mesh] OR "Attitude of Health Personnel"[Mesh] OR "Patient Discharge"[Mesh] OR "Decision Making, Shared"[Mesh] OR "Family Nursing"[Mesh] OR "Patient Discharge Summaries"[Mesh] | 1,316 |
| ProQuest Dissertation and Theses Global | mental illness* OR "severe mental illness*" OR "mental disorder*" OR "psychiatric disorder*" OR "psychiatric illness*" OR "mentally ill" OR "mental health condition*" OR "mental health disorder*" OR "mental health problem*" OR "mental health issue*" OR "psychiatrically ill" **AND** Emergency room* OR "emergency department*" OR "emergency health service*" OR "psychiatric hospital*" OR "acute care" OR "acute inpatient*" OR “Mental Health Service*” OR “Psychiatric Department, Hospital” OR “Involuntary Treatment, Psychiatric” OR “Psychiatric Nursing” **AND** Families OR "family member*" OR family OR parent* OR caregiver* OR carer* OR relative* OR spouse* OR "adult child*" OR partner* OR stakeholder* OR mother* OR sibling* OR sister* OR brother* OR father* OR wife OR wives OR husband* OR daughter* OR son OR sons OR grandparent* OR grandmother* OR grandfather* OR proxy OR proxies OR friend* OR boyfriend* OR "significant other*" OR girlfriend* OR elder* OR "loved one*" OR niece* OR nephew* OR cousin* OR uncle* OR aunt* **AND** Intervention* OR attitude* OR "discharge plan*" OR "shared decision making" OR "family centered care" OR "care plan*" OR treatment* OR need* OR involvement OR collaboration OR perception* OR perspective* OR impression* OR opinion* OR experience* OR "lived experience*" OR "health system response*" OR "health care system response*" OR "healthcare system response*" | 1,003 |
|  | **Total number of results:** | **22,970** |
